# Seminal Plasma Cytokines Are Predictive of the Outcome of Boar Sperm Preservation

**DOI:** 10.3389/fvets.2019.00436

**Published:** 2019-12-04

**Authors:** Isabel Barranco, Lorena Padilla, Cristina Pérez-Patiño, Juan M. Vazquez, Emilio A. Martínez, Heriberto Rodríguez-Martínez, Jordi Roca, Inmaculada Parrilla

**Affiliations:** ^1^Department of Medicine and Animal Surgery, Faculty of Veterinary Science, University of Murcia, Murcia, Spain; ^2^Department of Biology, Faculty of Sciences, University of Girona, Girona, Spain; ^3^Department of Clinical and Experimental Medicine (IKE), University of Linköping, Linköping, Sweden

**Keywords:** seminal plasma, cytokines, spermatozoa, liquid storage, cryopreservation, pig

## Abstract

**Background:** Boar seminal plasma is rich in cytokines, which could influence the capability of spermatozoa to tolerate preservation.

**Objectives:** To evaluate the involvement of boar seminal plasma cytokines in the changes experienced by boar spermatozoa during their storage, either in liquid or frozen state.

**Materials and Methods:** In two separated experiments, semen samples from healthy and fertile boars were split in two aliquots, one centrifuged twice (1,500 ×g for 10 min) to harvest seminal plasma, whereas the other was either commercially extended (3 × 10^7^ sperm/mL) and liquid-stored at 17°C during 144 h (*n* = 28, Experiment 1) or frozen-thawed using a standard 0.5 mL protocol (*n* = 27, Experiment 2). Sixteen cytokines were quantified using Luminex xMAP®. Sperm attributes (CASA-evaluated total and progressive motility; flow cytometry-evaluated sperm viability, production of intracellular H_2_O_2_ and ${\text{O}}_{2}^{•-}$ and levels of lipid peroxidation in viable spermatozoa) were evaluated either at 0, 72, or 144 h of liquid storage (Experiment 1) or before freezing and at 30- and 150-min post-thawing (Experiment 2).

**Results:** Multiple linear regression models, with Bayesian approach for variable selection, revealed that the anti-inflammatory TGF-β2, TGF-β3, IL-1Ra, and IL-4 and the pro-inflammatory IL-8 and IL-18, predicted changes in sperm motility for liquid-stored semen while the anti-inflammatory IFN-γ was included in the models predicting changes in all sperm attributes for cryopreserved semen.

**Conclusion:** Specific boar seminal plasma cytokines would contribute to modulate the structural and metabolic changes shown by spermatozoa during preservation, either in liquid or frozen state.

## Introduction

Cytokines comprise a family of ubiquitous low-molecular weight proteins released mainly by immune cells, mediating intercellular regulation of multiple cellular functions (1). Cytokines are also produced by a wide variety of cells in both male and female genital tracts, which are released to genital fluids (2–6). As such, a repertoire of cytokines has been identified in seminal plasma (SP) of humans (7), and other several mammalian species (8–11), including pigs, with quantitative variation among ejaculates (12). A balanced setup of SP-cytokines seems to intervene in the immediate and long-lasting immune responses of the female genital tract toward spermatozoa, embryos, and placentae, conditioning pregnancy success (13, 14). As well, unbalanced concentrations of SP-cytokines have been related with reproductive disorders in men (15), including infertility (5, 16).

Some seminal cytokines in human ejaculates appear to negatively affect sperm (17, 18), viability (19), or sperm capacitation (20). Similar studies relating SP-cytokines and sperm attributes in livestock are surprisingly few (8, 21) and fewer are even those studies assessing such relationship in semen preserved for artificial insemination (AI) purposes. To the best of our knowledge, only two studies have addressed this topic, focusing on particular cytokines. Specifically, Rodriguez-Gil et al. (21) highlighted the relevance of granulocyte macrophage colony-stimulating factor (GM-CSF) for the freezing of ovine spermatozoa while Mrkun et al. (22) searched for the effects of tumor necrosis factor-α (TNF-α) in the sperm quality of short-term liquid-stored porcine semen. Recent findings, indicating that boar SP is rich in cytokines but with concentrations differing substantially between mature boars routinely used in AI programs (12), provides the relevant pre-requisite to evaluate whether these SP-cytokines would affect the capacity of boar spermatozoa to sustain preservation, either at liquid- or at frozen state.

Artificial insemination is a routinely reproductive technology in the swine industry, currently focused on achieving the highest efficiency in fertility by using either liquid-stored or frozen-thawed semen doses (23, 24). It is well-known that sperm undergo injuries during storage, either at liquid or at frozen state, leading to a decline in sperm quality and functionality and, thereby, also in fertility (25, 26). To find out which factors affect the capability of porcine spermatozoa to tolerate preservation remains a challenge (27). Accordingly, the present study aimed to evaluate whether the relative concentrations of SP-cytokines in ejaculates collected from mature, healthy, and fertile boars used in AI-programs, could have a predictive value for the outcome of sperm preservation, after either extension and liquid storage (Experiment 1) or following freezing-thawing (Experiment 2).

## Materials and Methods

### Reagents and Media

All chemicals used in the experiments were of analytical grade and, unless stated otherwise, from Sigma-Aldrich (St. Louis, MO, USA). The media were prepared under sterile conditions in a laminar flow chamber (MicroH; Telstar, Terrassa, Spain). The probes C11-Borondipyrromethene 581/591 (BODIPY), 5-(and-6) chloromethyl-20,70-dichlorodihydrofluorescein diacetate acetyl ester (CM-H_2_DCFDA), dihydroethidium (DHE), SYTOX™ Green (SYTOX), and propidium iodide (PI) were from Invitrogen™ (Thermo Fisher Scientific, Waltham, MA, USA).

The basic medium used for semen extension was Beltsville Thawing Solution (BTS: 205 mM glucose, 20.4 mM sodium citrate, 10.0 mM KCl, 15.0 mM NaHCO_3_, 3.6 mM EDTA, and 0.05 mM kanamycin sulfate; pH 7.2–7.4, 290–300 mOsmol/kg). Phosphate-Buffered Saline EDTA-free (PBS: 139 mM NaCl, 2.7 mM KCl, 1.5 mM KH_2_PO_4_, and 8.1 mM Na_2_HPO_4_·7H_2_O with 0.058 g/L penicillin G and 0.05 g/L streptomycin sulfate; pH 7.0–7.2, 285–290 mOsmol/kg) was the medium used for flow cytometry analyses. The composition of the basic freezing medium (FM) was 80% (v/v) Tris-citric acid-glucose extender (111 mM Trizma Base, 31.4 mM monohydrate citric acid and 185 mM glucose with 100 μg/mL kanamycin sulfate; pH 7.0–7.4, 295–300 mOsmol/kg) with 20% (v/v) of egg yolk.

### Boars, Ejaculates and Seminal Plasma

Entire ejaculates were collected using a semi-automatic collection method (Collectis®, IMV Technologies, L'Aigle, France) from sexually mature and healthy boars (2–3 years old) of different breeds (Landrace, Large White, and Pietrain) included in AI-programs (Topigs Norsvin España, Madrid, Spain).

Seminal plasma was harvested after double centrifugation at 1,500 ×g for 10 min (Rotofix 32 A; Hettich Zentrifugen, Tuttlingen, Germany) of 15 mL semen samples, immediately after ejaculation. The second supernatant was microscopically examined (Eclipse E400; Nikon, Tokyo, Japan) to ensure it was sperm-free. The final SP samples were individually sent in insulated containers (15–17°C) to the Andrology Laboratory at the Veterinary Teaching Hospital of the University of Murcia within 2–3 h after ejaculate collection. At the laboratory, the SP-samples were frozen to −80°C (Ultra-Low Freezer; Haier, Qingdao, China) until cytokine quantification.

### Measurement of Cytokine Concentrations in Seminal Plasma

Cytokine concentrations in SP were quantified using the Luminex xMAP® technology, a multiplexed microsphere-based flow cytometric assay, following the protocols described by the manufacturers for 96-well multiscreen plates. Pre-coated magnetic beads (Cat#PCYTMG-23K-13PX for pig reactivity, Merck Millipore, Burlington, MA, USA) were used for the determination of GM-CSF, interferon-gamma (IFN-γ), interleukin (IL)-1α, IL-1β, IL-1Ra, IL-2, IL-4, IL-6, IL-8, IL-10, IL-12, IL-18, and TNF-α. The concentrations of transforming growth factor (TGF)-β1, -β2, and -β3 were measured using a 3-plex kit (Cat#TGFB-64K-03 for pig, human, mouse, rat, non-human primate, canine, feline reactivity, Merck Millipore) in SP-samples pre-acidified with 8 μL of HCl, extended with sample diluent (1/30, v/v) before processed as detailed below. A standard curve of 6 (TGFB-64K-03) or 7 (PCYTMG-23K-13PX) standard points, was built for each cytokine. Serum matrix (provided in the kits) was added in the standard, controls, and blank wells in order to mimic the SP-composition. Two controls (provided in the kits) were added as singlets in their corresponding wells. Bead solution was sonicated and added to each well and the plates were incubated at 4°C in the dark, overnight (16–18 h). After this period, the plates were emptied and washed twice (TGFB-64K-03) or thrice (PCYTMG-23K-13PX) with the washing solution provided in the kits. Each detection antibody was added to each well and the plates were incubated at room temperature (RT) in the dark for 60 min (TGFB-64K-03) o for 120 min (PCYTMG-23K-13PX). Thereafter, streptavidin–phycoerythrin was added to each well and the plates were incubated at RT for 30 min. After washing, the plates were run on a MAGPIX® (Luminexcorp, Austin, TX, USA) with xPONENT software version 4.2 (Luminexcorp) for acquisition and MILLIPLEX® Analyst Version 5.1 (Merck Millipore) for data analysis. The median fluorescent intensity was analyzed using a 5-parameter logistic curve-fitting to calculate the concentrations of the cytokines in each sample. All cytokine concentrations were expressed as pg/mL.

### Semen Preservation

#### Liquid Storage at 17°C

Once sperm concentration and sperm motility were quantified in the freshly collected ejaculates by using AI-center routinely analytical procedures, these were extended to 3 × 10^7^ sperm/mL using a commercial extender (Biosem+, Magapor, Zaragoza, Spain) and packed into 40 mL semen bags (Blue collection cone, IMV Technologies), which were stored in a cooled incubator at 17°C (FOC 120E Cooled Incubator; VELP Scientifica, Usmate Velate, Italy).

#### Cryopreservation

Samples of ejaculates were extended in BTS (1:1, v/v) immediately after ejaculation and stored at 17°C (FOC 120E Cooled Incubator) for 24 h. The samples were thereafter centrifuged (Megafuge 1.0 R, Heraeus, Hanau, Germany) at RT (3 min at 2,400 ×g) and the resulting sperm pellets were frozen using the 0.5-mL straw freezing procedure described by Alkmin et al. (28). Briefly, the pellets were extended in FM to a concentration of 1,500 × 10^6^ sperm/mL and cooled to 5°C for 150 min. Then, the samples were re-extended in FM supplemented with glycerol and Equex (89.5% FM + 1.5% Equex STM [v/v, Nova Chemical Sales, Scituate, MA, USA] and 9% glycerol [v/v; pH 6.2, 1,700–1,730 mOsmol/kg]) to a final concentration of 1,000 × 10^6^ sperm/mL. The extended sperm samples were packed into 0.5-mL polyvinyl chloride French straws (Minitüb, Tiefenbach, Germany) and frozen a commercial freezing unit (Minitüb, reference 15043/0736), of isolating styrofoam box with straw-bearing floating rack on the phase surface of liquid nitrogen (LN_2_) vapors (at 3 cm above LN_2_ surface for 20 min), routinely used for semen freezing. The frozen straws were stored in a LN_2_ container (GT40, Air Liquide, Paris, France) and remained there for at least 1 week before thawing, which was performed in a circulating water bath at 37°C for 20 s.

### Sperm Assessment of Processed Samples

Sperm attributes were assessed according to motility (total and progressive), integrity of plasma and acrosomal membranes (sperm viability) and the intracellular H_2_O_2_ generation, total ${\text{O}}_{2}^{•-}$ production and lipid peroxidation (LPO) in viable spermatozoa. Motility was objectively evaluated using a computer assisted sperm analyzer (CASA, ISASV1®, Proiser R+D S.L., Paterna, Spain). The other sperm attributes were cytometrically assessed using a BD FACS Canto II cytometer (Becton Dickinson Co, Franklin Lakes, NJ, USA). For this, Hoechst 33342 (H-42) dye was used to identify sperm events, with acquisition being stopped after 10,000 H-42 positive events.

To assess sperm motility, a pre-warmed (38°C) Makler counting chamber (Sefi Medical Instruments Ltd., Haifa, Israel) was loaded with 5 μL of extended semen (3 × 10^7^ sperm/mL in BTS) and a minimum of 400 spermatozoa per sample were microscopically analyzed (200×; UB200i, Proiser R+D S.L). Data were recorded as percentage of total motile spermatozoa (average path velocity ≥ 20 μm/s) and the proportion of motile spermatozoa showing rapid and progressive movement (straight line velocity ≥ 40 μm/s).

To assess the integrity of plasma and acrosomal membranes (sperm viability), 100 μL of semen sample (3 × 10^7^ sperm/mL in BTS) was mixed with 3 μL of H-42 (0.05 mg/mL in PBS), 2 μL of PI (0.5 mg/mL in PBS) plus 2 μL of fluorescein-conjugated peanut agglutinin (PNA-FITC, 100 μg/mL in PBS) and incubated at 38°C in the dark for 10 min. Thereafter, stained sperm samples were extended in PBS to reach 3 × 10^6^ sperm/mL and cytometrically analyzed. Data were recorded as the percentage of viable spermatozoa with intact acrosome, namely those H-42 positive and PI and PNA-FITC negative.

Intracellular H_2_O_2_ generation was measured in viable spermatozoa (H-42 positive and PI negative) using CM-H_2_DCFDA dye and following with slight modifications the procedure described by Guthrie and Welch (29). Briefly, 50 μL of semen sample (3 × 10^7^ sperm/mL in BTS) was mixed with 1.25 μL of H-42 (0.05 mg/mL in PBS), 1 μL of PI (0.5 mg/mL in PBS) plus 1 μL of CM-H_2_DCFDA [1 mM in dimethyl sulfoxide (DMSO)], extended in 950 μL of PBS and incubated at 38°C in the dark for 30 min. A similar semen sample, including 1 μL of tert-butyl hydroperoxide (TBH) solution (70% in distilled water), was used as positive control. The percentage of viable (H-42 positive and PI negative) spermatozoa positive to 2′,7′-di-chlorofluorescein were recorded as H_2_O_2_ generation.

Total sperm ${\text{O}}_{2}^{•-}$ production was assessed using DHE following a modification of the procedure described by Koppers et al. (30). Briefly, 1 mL of semen sample (1 × 10^7^ sperm/mL in BTS) was mixed with 10 μL of SYTOX (5 μM in DMSO), 15 μL of H-42 (0.05 mg/mL in PBS) plus 10 μL of DHE (200 μM in DMSO) and incubated in the dark for 15 min at 37°C. A similar semen sample, including 10 μL of TBH solution (70% in distilled water), was used as a positive control. Thereafter, the semen samples were centrifuged (5 min at 600 ×g at RT) and the sperm pellets were re-extended in PBS to reach a 1 mL sample. Before flow cytometry analysis, semen samples were further re-extended in PBS to reach 3 × 10^6^ sperm/mL. The percentage of viable spermatozoa (H-42 positive and SYTOX negative) positive to DHE were recorded as total ${\text{O}}_{2}^{•-}$ production.

The LPO was assessed using BODIPY following a modification of the procedure described by Koppers et al. (30). Briefly, 1 mL of semen sample (2 × 10^7^ sperm/mL in BTS) was mixed with 2.5 μL of BODIPY (2 mM in ethanol) and incubated for at 37°C in the dark for 30 min. The semen samples were centrifuged (300 ×g for 7 min at RT) and the sperm pellets were extended in 1 mL of PBS. Then, 100 μL of each semen sample was mixed with 2 μL of H-42 (0.05 mg/mL in PBS) plus 1.3 μL of PI (0.5 mg/mL in PBS) and incubated at 37°C in the dark for 10 min. A similar semen sample, including 10 μL of TBH solution (70% in distilled water), was used as positive control. Before flow cytometry analysis, semen samples were re-extended in PBS to reach 3 × 10^6^ sperm/mL. The percentage of viable spermatozoa (H-42 positive and PI negative) positive to BODIPY were recorded as LPO.

### Experimental Design

The study pursued to explore predictive relationships between the concentrations of cytokines in boar ejaculate SP and the changes in sperm attributes following extension and liquid-storage (as per AI-doses, Experiment 1) or cryopreservation in straws (Experiment 2).

For the Experiment 1, a total of 28 entire ejaculates (one per boar) were collected. All ejaculates fulfilled the standards of quantity (>200 × 10^6^ sperm/mL) and sperm quality parameters (80% viability, 75% motility, and 80% depicting normal morphology) required for the preparation of AI-semen doses. Samples of each ejaculate were split in two aliquots; one was used to obtain SP samples for cytokine concentration analyses. The other one was extended to 1.2 × 10^9^ spermatozoa/40 mL dose and stored at 17°C for 144 h. The average proportion of SP in each extended semen dose was approximately 30%, ranging from 25 to 33%.

For Experiment 2, a total of 27 entire ejaculates (one per boar) were split in two aliquots. One was used to obtain SP samples for cytokine concentration analyses and the other one was cryopreserved following the protocol described above. Since semen samples were stored at 17°C during 24 h after extension 1:1 (vol/vol) in BTS, the spermatozoa remained surrounded with their own SP at 50% concentration. Sperm attributes were assessed before freezing (Control) and after thawing, following re-extension in BTS (500 × 10^6^ sperm/mL), at 30 and 150 min of incubation at 37°C.

### Statistical Analysis

Data were analyzed using IBM SPSS Statistics 19.0 (IBM Spain, Madrid) and R software (www.r-project.org). The Shapiro-Wilk test was used to check the assumption of normality of data of sperm parameters and SP-cytokine concentrations. The influence of liquid storage time (Experiment 1), freezing-thawing and post-thaw incubation time (Experiment 2) over sperm attributes was evaluated using one-way ANOVA. The Bonferroni test was used for *post-hoc* analyses where appropriate. Statistical significance was defined as *p* < 0.05. Results of quality and sperm functionality were showed as mean ± standard error of the mean (SEM).

Multiple linear regression models were performed to evaluate the influence of SP-cytokines and time (liquid storage time or frozen-thawing and post-thawing incubation time) on sperm parameters. Before running regression analyses, BayesVarSel package of R was used, following the recommendations given by Bayarri et al. (31) for testing both the objective hypotheses and the selection of variables. Thereafter, the correlation among selected predictor variables was evaluated in order to identify and remove those showing co-linearity and, consequently, avoiding redundancy between the selected predictor variables. A correlation coefficient >0.7 was used as discriminating. The linear relationship between the dependent variables and the predictor variables and the homoscedasticity of residuals were checked to validate the strength of the multiple linear regression models chosen.

## Results

### Cytokine Concentrations in Seminal Plasma

The concentrations of the battery of cytokines measured in the SP-samples of boar ejaculates used for storage as liquid-extended samples (Experiment 1) or frozen (Experiment 2) are shown in Table 1. The TGF-β2, IFN-γ, TGF-β1, and IL-1Ra are, in this order, the cytokines showing the highest concentrations whereas the concentrations of IL-12, IL-10, and IL-1α were, in this order, the lowest. The concentration of IL-12 was under the limits of detection in 52 of the 55 SP-samples evaluated (only three of the 28 SP-samples from liquid-stored semen samples showed measurable levels). A similar situation occurred with IL-1β.

**Table 1:** Cytokine concentrations (median and interquartile range, IQR) in the seminal plasma of boar semen samples either liquid-stored at 17°C (Experiment 1) or cryopreserved (Experiment 2).

**Cytokines^a^ (pg /mL)**	**Seminal plasma**			
	**Liquid-stored semen (Experiment 1**, ***n*** **=** **28)**		**Frozen-thawed semen (Experiment 2**, ***n*** **=** **27)**	
	**Median**	**IQR**	**Median**	**IQR**
TGF-β1	3,578	2,268	1,785	1,558
TGF-β2	33,255	34,707	20,406	20,833
TGF-β3	283	195	263	242
GM-CSF	70	45	86	37
IFN-ɤ	5,023	7,604	4,169	7,039
IL-1a	9	12	8	9
IL-1β	106^*^	0	106^*^	0
IL-1Ra	581	900	990	2,622
IL-2	34	36	24	19
IL-4	22	10	22	7
IL-6	45	82	6^*^	5
IL-8	64	110	46	28
IL-10	8	6	8	5
IL-12	5^*^	0	5^*^	0
IL-18	52	49	57	49
TNF-α	57	69	46	42

a *Cytokines: Transforming growth factor (TGF), growth factor granulocyte–macrophage colony-stimulating factor (GM-CSF), interferon (IFN), interleukin (IL), and tumor necrosis factor (TNF)*.

* *Lower limit of detection*.

### Quality and Sperm Functionality Parameters

Liquid storage time at 17°C (Experiment 1) influenced some sperm attributes (Table 2). Sperm motility, measured in terms of total and progressive motility, declined (*p* < 0.001) between 72 and 144 h of storage while sperm viability was not affected by storage time. The generation of ROS (measured in terms of H_2_O_2_ and total ${\text{O}}_{2}^{•-}$ production) and LPO by viable spermatozoa progressively increased along storage time (*p* < 0.01). Yet, the percentages of viable spermatozoa showing both a high production of ${\text{O}}_{2}^{•-}$ and LPO were low in the three storage times, always below 3 and 2%, respectively. Freezing-thawing and post-thawing incubation time at 37°C affected all sperm parameters evaluated (Table 3). Total and progressive motility and viable sperm with intact acrosome declined (*p* < 0.01) and generation of ROS (measured in terms of total ${\text{O}}_{2}^{•-}$ and H_2_O_2_ production) and of LPO by viable spermatozoa increased (*p* < 0.001).

**Table 2:** Sperm attributes in boar semen liquid-stored at 17°C during 144 h (*n* = 28).

**Sperm parameter (%)**	**Storage time at 17^°^C**			***P*-value**
	**0 h^2^**	**72 h**	**144 h**	
Total motility	81.11 ± 2.06^a^	72.64 ± 2.70^a^	48.57 ± 5.67^b^	<0.001
Progressive motility	44.46 ± 2.73^a^	46.36 ± 2.59^a^	30.11 ± 4.30^b^	0.001
Viable sperm with intact acrosome	90.96 ± 1.01	88.31 ± 1.25	86.10 ± 1.97	ns
H_2_O_2_ generation^1^	5.30 ± 0.66^a^	8.16 ± 0.58^a^	25.39 ± 1.97^b^	<0.001
Total ${\text{O}}_{2}^{•-}$ production^1^	0.64 ± 0.15^a^	1.95 ± 0.23^b^	2.78 ± 0.40^b^	<0.001
Lipid peroxidation^1^	0.42 ± 0.10^a^	0.97 ± 0.20^a^^,^^b^	1.41 ± 0.31^b^	0.008

1 *Viable spermatozoa*;

2 *Assessed at 2–4 h after ejaculate collection*.

a, b *Indicates significant differences among storage times within the same sperm parameter*.

**Table 3:** Sperm attributes in frozen-thawed boar semen samples (*n* = 27).

**Sperm parameter (%)**	**Fresh semen^2^**	**Post-thaw evaluation time**		***P*-value**
		**30 min**	**150 min**	
Total motility	75.82 ± 2.32^a^	39.93 ± 3.79^b^	25.81 ± 3.24^c^	<0.001
Progressive motility	28.48 ± 2.64^a^	30.78 ± 3.24^a^	18.59 ± 2.69^b^	0.008
Viable sperm with intact acrosome	78.24 ± 2.73^a^	41.46 ± 3.25^b^	36.78 ± 3.24^c^	<0.001
H_2_O_2_ generation^1^	3.93 ± 0.89^a^	8.82 ± 1.53^b^	10.71 ± 0.72^b^	0.001
Total ${\text{O}}_{2}^{•-}$ production^1^	1.86 ± 0.26^a^	16.26 ± 1.86^b^	24.91 ± 3.19^c^	<0.001
Lipid peroxidation^1^	1.32 ± 0.56^a^	12.03 ± 2.45^b^	16.98 ± 3.01^c^	<0.001

1 *Viable spermatozoa*;

2 *Assessed at 16–24 h after ejaculate collection in semen samples diluted 1:1 (vol/vol)*.

a, b, c *Indicates significant differences among the evaluation times within the same sperm parameter*.

### Seminal Cytokines as Predictors for Sperm Quality and Functionality

A series of multiple linear regression models was generated considering all possible combinations for the sperm attributes affected by preservation. Those with more predictive weight are summarized in Table 4 (Experiment 1, liquid-stored semen) and Table 5 (Experiment 2, frozen-thawed semen). For liquid-stored semen (Table 4), the predictive value of the seminal cytokines differed according to storage time at 17°C, being significant (*p* < 0.05) only by 144 h of storage. At this storage time, the seminal cytokines showed good predictive value (adjusted R^2^ equal or above 60%) for total and progressive motility, as they explained 68 and 60% of total variance, respectively. The predictive value of seminal cytokines was lower (adjusted R^2^ below 40%) for the other sperm parameters evaluated. Seven SP-cytokines were selected in the regression models for motility parameters, six of them common for total and progressive motility, specifically the anti-inflammatory TGF-β2, TGF-β3, IL-1Ra, and IL-4 and the pro-inflammatory IL-8 and IL-18.

**Table 4:** Prediction values (±standard error) of linear regression models estimating how levels of seminal plasma cytokines foresee changes (negative or positive) of sperm attributes in liquid-stored boar semen.

**Seminal cytokines^a^**	**Sperm attributes**				
	**Total motility**	**Progressive motility**	**H_**2**_O_**2**_ generation^b^**	**Total O_**2**_ generation^b^**	**Lipid peroxidation^b^**
TGF-β1					−1.08 ± 0.32
TGF-β2	−1.60 ± 0.28	−1.30 ± 0.37			
TGF-β3	2.91 ± 0.45	2.53 ± 0.58	0.14 ± 0.03		
GM-CSF					
IFN-γ			0.09 ± 0.02		
IL-1α					
IL-1β					
IL-1Ra	−1.01 ± 0.29	−1.40 ± 0.38			
IL-2					
IL-4	−274.57 ± 36.39	−297.36 ± 41.61			
IL-6					
IL-8	4.17 ± 0.94	6.75 ± 1.17			
IL-10	139.09 ± 57.77				
IL-12		−157.78 ± 44.89	−4.84 ± 1.07	−96.32 ± 39.35	
IL-18	22.74 ± 7.27	44.62 ± 8.77			
TNF-α					
*F*-value	8.28	6.14	7.42	7.10	4.26
Adjusted *R*^2^	0.68	0.60	0.39	0.35	0.17
*P*-value	<0.001	<0.001	<0.001	<0.001	0.002

a *Seminal cytokines: Transforming growth factor (TGF), growth factor granulocyte–macrophage colony-stimulating factor (GM-CSF), interferon (IFN), interleukin (IL), and tumor necrosis factor (TNF)*.

b *Viable sperm*.

**Table 5:** Prediction values (±standard error) of linear regression models estimating how levels of seminal plasma cytokines foresee changes (negative or positive) of sperm attributes in frozen-thawed boar semen.

**Seminal cytokines^a^**	**Sperm attributes**					
	**Total motility**	**Progressive motility**	**Viable sperm with intact acrosome**	**H_**2**_O_**2**_ generation^b^**	**Total O_**2**_ generation^b^**	**Lipid peroxidation^b^**
TGF-β1	−0.69 ± 0.24	−1.15 ± 0.28				2.45 ± 0.28
TGF-β2						
TGF-β3						
GM-CSF				30.48 ± 12.91		
IFN-γ	1.21 ± 0.17	1.65 ± 0.18	0.68 ± 0.11	1.32 ± 0.48	−0.42 ± 0.10	−0.27 ± 0.18
IL-1α			57.58 ± 11.60	−72.41 ± 34.22		
IL-1β						
IL-1Ra	−0.80 ± 0.19	−1.05 ± 0.21	−1.21 ± 0.20			−0.56 ± 0.21
IL-2	−21.69 ± 9.48					
IL-4		−113.11 ± 30.60	−68.33 ± 18.72	−246.29 ± 116.79		
IL-6					10.69 ± 2.82	
IL-8	−17.24 ± 4.91	−22.14 ± 5.36	−15.17 ± 3.17		9.89 ± 4.02	20.05 ± 5.33
IL-10					176.04 ± 28.05	
IL-12						
IL-18					−17.72 ± 4.60	
TNF-α						
*F*-value	15.15	22.08	22.69	70.09	14.58	22.18
Adjusted *R*^2^	0.63	0.70	0.67	0.60	0.61	0.62
*P*-value	<0.001	<0.001	<0.001	<0.001	<0.001	<0.001

a *Seminal cytokines: Transforming growth factor (TGF), growth factor granulocyte–macrophage colony-stimulating factor (GM-CSF), interferon (IFN), interleukin (IL), and tumor necrosis factor (TNF)*.

b *Viable sperm*.

For frozen-thawed semen (Table 5), the post-thaw incubation time influenced the outcome, but being additive to that of the SP-cytokines. The predictive value of the seminal cytokines on post-thaw sperm attribute changes differed from that shown for liquid-stored semen, involving other SP-cytokines. Here the SP-cytokines showed good predictive value (adjusted R^2^ above 60%) for all sperm parameters evaluated. The regression models included between four and five SP-cytokines, being the anti-inflammatory IFN-γ the only present in all regression models.

## Discussion

The present study confirmed that the porcine SP is rich in cytokines (12) but, more importantly, that the levels of particular SP-cytokines appear predictive of the capability of spermatozoa to sustain preservation, either at liquid or frozen state.

The hereby tested SP of mature, healthy, fertile, and sexually active boars, routinely used in AI-programs, contained variable concentrations of a broad set of cytokines, with pro- or anti-inflammatory properties. These results not only agree with previous findings in pig SP (12) but also those in healthy males of other mammalian species, including human (32, 33). In addition, in agreement with previous studies in SP of boar (12) and men (34), the results also clearly show that cytokine SP-levels varied greatly among semen samples. Overall, the findings imply that cytokines are a physiological constituent of SP and, therefore, their presence is not only linked to functional reproductive disorders, as most broadly considered in studies where they were used as diagnostic markers, but perhaps influencing sperm function in fertile individuals, when semen is processed for AI, as an example.

The present study also confirmed that the sperm attributes of motility, membranes intactness, ROS production and degree of LPO were affected by the standard preservation procedures used, either at liquid or frozen state, as expected and agreed with that reported in previous studies (35–37). The results further clearly demonstrated that boar spermatozoa are more sensitive to freezing than to liquid storage, where spermatozoa only showed some biologically relevant changes when stored for up to 144 h. So, while all the evaluated sperm parameters were clearly disturbed by freezing storage, only motility parameters and H_2_O_2_ generated by viable spermatozoa experienced changes with biological relevance during liquid storage. Accordingly, the regression models generated for evaluating the relevance of SP-cytokines on sperm preservation seemed sound for all the parameters evaluated in the frozen-thawed spermatozoa but only for total and progressive motility in the liquid-stored spermatozoa.

To generate the regression models, the cytokines that should be included in the models were selected using a Bayesian approach, following the recommendations of Ranciati et al. (38). According to these authors, Bayesian approaches are suitable because they allow to quantify the relevance of the variables together with the handling of not normally distributed data. Remarkable was the large number of cytokines selected by the regression models. Specifically, 9 and 10 cytokines, respectively, explained changes registered in sperm attributes relevant for survival and function when stored at either liquid or frozen state. Worth to note was also the fact that the cytokines selected to explain the changes experienced by a specific sperm attribute during or post-preservation were not equal for liquid-stored or frozen-thawed spermatozoa. This finding further supports the well-founded theory that pig spermatozoa react differently to the stress experienced during liquid storage than during freezing and thawing, the latter causing different and more severe disorders (26).

Many of the selected cytokines in the regression models are signaling molecules involved in pathways regulating sperm functions. For instance, the mitogen-activated protein kinase (MAPK) or the tyrosine kinase (JAK/STAT) (15, 20, 39), are both closely related with sperm motility (40) and the TGFβs IFN-γ, and IL-6 would be among the cytokines involved in these pathways (39). Focusing on IFN-γ, its SP concentrations were also found positively related to increased sperm membrane permeability, linked to LPO, and negatively related with sperm motility in human ejaculates (41, 42). Boar spermatozoa have receptors to IFN-γ (43) and the concentration measured in boar SP was higher than that reported in SP of healthy men (33). Maybe this would explain why IFN-γ was the only SP-cytokine present in all generated regression models explaining the registered changes in sperm attributes following freezing and thawing. Another cytokine involved in the above signaling pathways is IL-6, whose SP-levels in men were also positively related with a greater ROS generation by spermatozoa (44). Accordingly, IL-6 was one of the SP-cytokines selected in the regression model to explain the production of ${\text{O}}_{2}^{•-}$ by cryosurviving boar spermatozoa. The pathways that other selected cytokines, as IL-4 or IL-8, would use for explaining changes in sperm attributes during preservation remains to be described. Abnormal SP levels of these cytokines have been related with changes in sperm quality (45) and with infertility (46) in human. Seminal pro-inflammatory cytokines, such as IL-8, IL-12, and IL-18, would play a key role in regulating sperm oxidative status and, thereby, they would influence sperm performance (44, 47–51). In this context, Fraczek et al. (52) demonstrated that SP-IL-8 enhances membrane LPO in human spermatozoa.

The above background would support the involvement of many of the selected SP-cytokines in the changes in quality and functionality experienced by boar spermatozoa during preservation, either at liquid or frozen state. However, recent evidence supports the concept that cytokines, including those in SP, operate more as a network than individually (53), being the existing interactions among them currently under unveiling (11). A functional cytokine network would be a way to understand the involvement of IL-12 on the changes in progressive motility experienced by boar spermatozoa during liquid storage, as the measured SP concentrations of IL-12 were almost always baseline, with measurable levels only in a small number of semen samples. In this regard, it has been indicated that disorders in SP concentration of IL-12 and IL-18, together but not individually, increased DNA fragmentation in human spermatozoa (54). Interestingly, both cytokines, IL-12 and IL-18, were selected by the regression model to explain changes in progressive motility experienced by liquid-stored spermatozoa. In the context of cytokine networks, it is also appropriate to highlight that IFN-γ has the ability to activate several other cytokines, such as IL-1Ra and IL-18 (55). Maybe such a network could explain why IFN-γ and IL-1Ra were two of the selected cytokines for explaining the changes experienced by cryosurvival spermatozoa in total and progressive motility, viability and LPO.

Seminal cytokines would influence sperm functionality after binding to specific sperm receptors, as demonstrated for instance for IL-6 (17). Therefore, to understand how SP-cytokines influence the intactness of structural and metabolic sperm parameters of preserved spermatozoa, it is essential to know the dynamic flow experienced by cytokine receptors during sperm preservation and the ability of SP-cytokines to bind to them. Unfortunately, there are currently few studies focused on identifying specific cytokine receptors in mature spermatozoa (43, 56, 57). A matter of utmost relevance is the timing when such interactions actually occur. Are these levels of SP-cytokines found in the ejaculate brief? Or they are an expression of testicular, epididymal, and accessory gland contributions over an extended period? Or, are the spermatozoa further influenced by the aliquots of SP that are maintained when the fresh ejaculate is extended for liquid storage (usually about 30%) for 144 h or in a highest proportion (50%) maintained for 24 h before the spermatozoa are to be frozen? Either possibility is worth following, although most likely are many SP-cytokines bound to the spermatozoa while others are probably present in the SP for their ability to interact with the lining epithelium of the female internal genital tract. More studies are warranted to follow.

Another question arising from our results is why SP cytokines that influence the same sperm attribute (for example, total motility) would be different for liquid storage than for cryopreservation? Indeed, alike the previous questions, this is also open to further hypotheses. The management of spermatozoa substantially differs between both conservation procedures, the processing for cryopreservation being more cell-disturbing because requires centrifugation that can stimulate the binding of SP proteins (perhaps cytokines among others) and/or the shedding of others (including cytokine receptors). Recently, we demonstrated that GM-CSF bound to boar sperm was higher in frozen-thawed spermatozoa than in spermatozoa stored in liquid state (58), as similarly demonstrated for other SP proteins (37). Furthermore, it is well-known that cryopreservation alters boar sperm membranes with greater intensity and in a different way than liquid storage (59). Therefore, it would be possible that the arrangement of cytokine receptors, and the subsequent SP-cytokine binding could both be affected in different ways during the processing, as has been recently demonstrated for other boar sperm membrane proteins (60).

In summary, this study reveals, for the first time, that many of the SP-cytokines hereby mapped would contribute to modulate the structural and metabolic changes shown by spermatozoa during preservation, differing between liquid-state storage, or frozen-thawed. At present, unfortunately, only a restricted number of pathways is known for some of the studied cytokines, while other relations between SP-levels and influence can only be speculated. In any case, it seems that the SP-cytokines work in networks rather than individually. In our opinion, this study shows valuable results that could be useful to understand the deterioration of structural and metabolic sperm parameters during preservation, with differences between liquid-storage and freezing-thawing. However, the limited number of semen samples preserved and evaluated is a reason for caution. Further studies, evaluating a larger number of semen samples from males showing clear differences in both their capability to sustain sperm preservation, at liquid and/or frozen state, and the SP concentration of cytokines should be performed to confirm the relevance of the present findings. The results of the present study also open for future studies evaluating cytokines or their antagonists as potential additives for extenders of pig semen.

## Data Availability Statement

The datasets generated for this study are available on request to the corresponding author.

## Ethics Statement

All procedures involving animals were performed according to international guidelines (Directive 2010/63/EU), following the approval of the Bioethics Committee of Murcia University (research code: 639/2012).

## Author Contributions

JR, JV, EM, and HR-M obtained the funding to carry out the study. JR, IP, and IB conceived and designed the study, analyzed, and interpreted the data. IB, LP, and CP-P performed the experiments. IB and LP wrote the draft manuscript. JR, JV, and HR-M revised and discussed the manuscript. All authors read and approved the manuscript for publication.

### Conflict of Interest

The authors declare that the research was conducted in the absence of any commercial or financial relationships that could be construed as a potential conflict of interest.
